# N-Acetylcysteine Suppresses Microglial Inflammation and Induces Mortality Dose-Dependently via Tumor Necrosis Factor-α Signaling

**DOI:** 10.3390/ijms24043798

**Published:** 2023-02-14

**Authors:** Mai Sakai, Zhiqian Yu, Masayuki Taniguchi, Rosanne Picotin, Nanami Oyama, David Stellwagen, Chiaki Ono, Yoshie Kikuchi, Ko Matsui, Miharu Nakanishi, Hatsumi Yoshii, Tomoyuki Furuyashiki, Takaaki Abe, Hiroaki Tomita

**Affiliations:** 1 Department of Psychiatric Nursing, Graduate School of Medicine, Tohoku University, Sendai 980-8575, Japan; 2 Department of Psychiatry, Graduate School of Medicine, Tohoku University, Sendai 980-8575, Japan; 3 Division of Pharmacology, Kobe University Graduate School of Medicine, Kobe 650-0017, Japan; 4 Medical Faculty, Heinrich Heine University Düsseldorf, 40225 Düsseldorf, Germany; 5 Department of Neurology and Neurosurgery, The Research Institute of the McGill University Health Center, Montreal, QC H3G 1A4, Canada; 6 Super-network Brain Physiology, Graduate School of Life Sciences, Tohoku University, Sendai 980-8577, Japan; 7 Department of Biomedical Engineering Regenerative and Biomedical Engineering Medical Science, Graduate School of Biomedical Engineering, Tohoku University, Sendai 980-8575, Japan; 8 Department of Disaster Psychiatry, International Research Institute for Disaster Science, Tohoku University, Sendai 980-8573, Japan

**Keywords:** microglia, LPS, N-acetylcysteine (NAC), nitrite and nitrate (NO_2_^−^NO_3_^−^), mortality

## Abstract

N-acetylcysteine (NAC) is an antioxidant that prevents tumor necrosis factor (TNF)-α-induced cell death, but it also acts as a pro-oxidant, promoting reactive oxygen species independent apoptosis. Although there is plausible preclinical evidence for the use of NAC in the treatment of psychiatric disorders, deleterious side effects are still of concern. Microglia, key innate immune cells in the brain, play an important role in inflammation in psychiatric disorders. This study aimed to investigate the beneficial and deleterious effects of NAC on microglia and stress-induced behavior abnormalities in mice, and its association with microglial TNF-α and nitric oxide (NO) production. The microglial cell line MG6 was stimulated by Escherichia coli lipopolysaccharide (LPS) using NAC at varying concentrations for 24 h. NAC inhibited LPS-induced TNF-α and NO synthesis, whereas high concentrations (≥30 mM) caused MG6 mortality. Intraperitoneal injections of NAC did not ameliorate stress-induced behavioral abnormalities in mice, but high-doses induced microglial mortality. Furthermore, NAC-induced mortality was alleviated in microglial TNF-α-deficient mice and human primary M2 microglia. Our findings provide ample evidence for the use of NAC as a modulating agent of inflammation in the brain. The risk of side effects from NAC on TNF-α remains unclear and merits further mechanistic investigations.

## 1. Introduction

Inflammation involves homeostasis and disease processes, including diabetes, cancer, heart disease, arthritis, neurological disease, and psychiatric disorders [1,2]. The relevance of inflammation in those conditions has been proposed to be linked with alterations of production in cytokines and reactive oxygen species (ROS) [1]. N-acetylcysteine (NAC) is an acetylated variant and precursor of the amino acid L-cysteine [3], has excellent effects on inflammation [4] and ROS [5], and has been used for decades as a nutritional supplement and low-cost medication for various ailments [6]. In the central nervous system (CNS), NAC is an important antioxidant [7] that promotes the inhibition of the nitric oxide synthase (iNOS) enzyme, resulting in a decrease in the production of tumor necrosis factor (TNF)-α and nitric oxide (NO) in astrocytes [8]. Furthermore, NAC is known to prevent apoptosis and oxygen-related genotoxicity by increasing intracellular levels of glutathione and decreasing mitochondrial membrane depolarization [5]. The properties of NAC include promoting neurogenesis [9] and ameliorating the inflammatory process [10] in various psychiatric disorders, including Alzheimer’s disease [11], anxiety [12], depression [13], and bipolar disorder [14]. Previous studies have demonstrated that NAC is effective as an adjunctive treatment and has a positive cognitive effect on several psychiatric disorders [15]. For instance, NAC improved symptoms of attention deficit hyperactivity disorder in patients with systemic lupus erythematosus [16]. Patients with bipolar disorder who received NAC experienced remission of depressive and manic symptoms [17]. In patients with chronic schizophrenia, a clinical trial combining NAC treatment with antipsychotic drugs improved the Positive and Negative Syndrome Scale scores [18]. Berk et al. found that NAC had limited efficacy as an adjunctive therapy for major depressive disorder (MDD) [19].

Microglia are primary immune cells in the brain that contribute to CNS development and homeostasis by removing apoptotic newborn neurons and pruning developing axons and synapses [20]. Lipopolysaccharide (LPS) induces microglia activation by releasing pro-inflammatory cytokines and NO [21,22], which promote ROS to trigger apoptosis [23,24,25]. NO, neuroinflammation-associated protein aggregation and neuronal damage have been shown to cause the activation of disease-associated microglia [26]. Excessive microglial NO release is a major contributor to neuronal death [27]. ROS are generated in TNF-α-mediated apoptosis after the exposure of cerebellar granule cells to NO donor S-nitroso-N-acetylpenicillamine [24]. Additionally, NO regulates the release of factors and hormones that are part of the neuroendocrine axis, allowing microglia to exert certain influences under immune-activating conditions [28]. It has also been shown that NO and its metabolites can induce therapeutic effects in various neurological disorders via mitochondrial functions [29]. In animal models, for instance, NAC pretreatment has been shown to alleviate pancreatitis by increasing serum NO levels [30].

NAC is known to prevent cell death by inhibiting ROS-induced oxidative stress. It reduces LPS- and plasminogen-induced microglial TNF-α and IL-1β production [31,32]. As demonstrated by several animal and clinical studies, TNF-α leads to neuronal damage and is associated with several psychiatric disorders, such as Alzheimer’s disease, Parkinson’s disease, autism spectrum disorder, MDD, schizophrenia, and posttraumatic stress disorder [21,33,34,35,36]. TNF-α may contribute to the pathogenesis of psychiatric disorders via hypothalamic-pituitary-adrenocortical axis activation, which leads to the immunologically mediated neurotoxic release of glutamate [37].

NAC can also act as pro-oxidants in the presence of transition metal ions such as Cu^2+^ or vitamin B12 [38,39]. NAC enhances fisetin-mediated but ROS-independent apoptosis in human colonic cancer cells [40]. Although NAC has been widely used safely in the clinical setting for decades, side effects of NAC in clinical treatment have been identified, most commonly gastrointestinal and dermatological disorders [41]. NAC treatment (10 mg/kg) for one week after eccentric exercise-induced muscle damage significantly increased tissue damage and oxidative stress compared to controls [42]. Chronic systemic NAC administration caused pulmonary arterial hypertension in mice [43]. Continuous infusion of high-dose NAC during a lipopolysaccharide challenge increased mortality in rats [44]. These effects probably occurred directly in the brain, as NAC crossed the blood–brain barrier and accumulated there [45]. However, the effective dose of NAC and its toxicity to microglial cells remain unknown.

We used microglial cells as a model system, which produce TNF-α and NO synthesis upon LPS stimulation. This study aimed to: (1) investigate the dose–response and time course of the effect of LPS on TNF-α and NO production, and (2) identify the beneficial and deleterious effects of NAC using varying doses that are commonly used in in vitro and in vivo research. Our findings highlighted the potential applications of NAC in the treatment of psychiatric diseases.

## 2. Results

### 2.1. Effect of LPS on Microglial Activation and Viability

**Figure 1:**
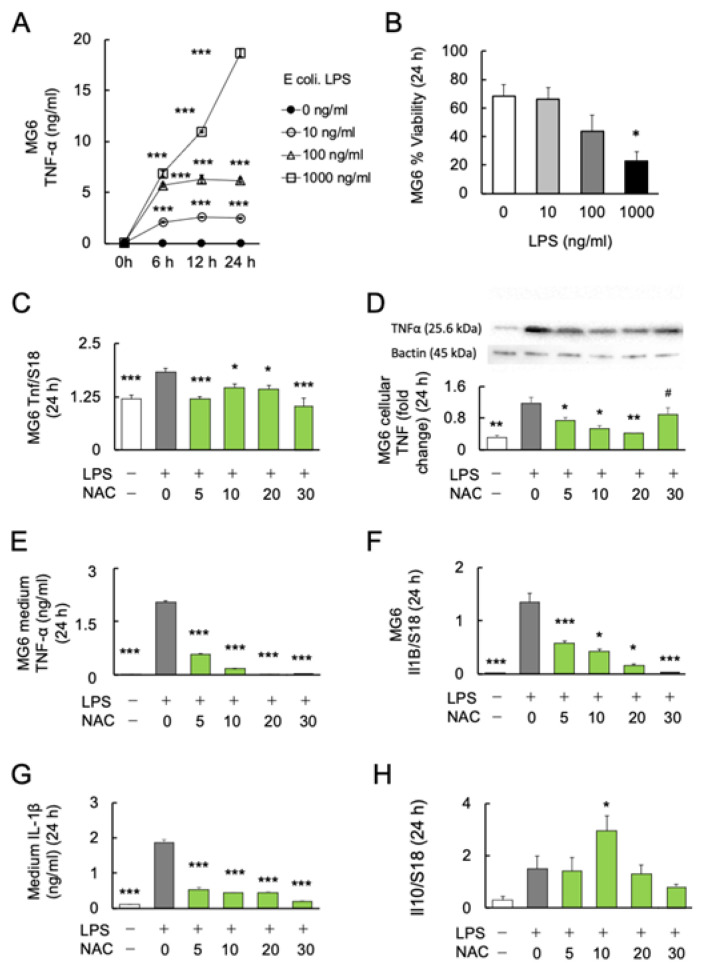
Effects of NAC on LPS-induced cytokines production in MG6 cells. (**A**) The dose–response relationship and time course of LPS on TNF-α production (n = 6). *** *p* < 0.001, vs. control. (**B**) The dose–response relationship of LPS with the viability of MG6 cells after 24 h (n = 6). * *p* < 0.05, vs. control. (**C**) The dose–response relationship of NAC with LPS-induced *Tnf* mRNA expression in MG6 cells after 24 h (n = 6). * *p* < 0.05, *** *p* < 0.001, vs. LPS only. (**D**) Gel images and bar graph of the signal intensities determined by western blotting of MG6 cells using anti-TNFα antibodies after 24 h treatment with LPS and NAC, relative to the averaged signal intensity of β-actin (n = 3). * *p* < 0.05, ** *p* < 0.01, vs. LPS only. # *p* < 0.05, vs. Con. (**E**) The dose–response relationship of NAC with LPS-induced TNF-α synthesis in MG6 cells after 24 h (n = 6). *** *p* < 0.001, vs. LPS only. (**F**) The dose–response relationship of NAC with LPS-induced *Il1b* mRNA expression in MG6 cells (n = 6). * *p* < 0.05, *** *p* < 0.001, vs. LPS only. (**G**) The dose–response relationship of NAC with LPS-induced IL-1 synthesis in the medium of MG6 cells (n = 6) after 24 h. *** *p* < 0.001, vs. LPS only. (**H**) The dose–response relationship of NAC with LPS-induced *Il10* mRNA expression in MG6 cells after 24 h (n = 6). * *p* < 0.05, vs. control. All data are presented as MEAN ± SEM.

To determine the dose–response relationship and time course of *E. coli* LPS-induced microglial TNFα production, MG6 cells were stimulated with 0, 10, 100, and 1000 ng/mL LPS over a time course of 0, 6, 12, and 24 h. The protein levels of the major inflammatory cytokine TNF-α in the MG6 culture supernatant were determined. Two-way ANOVA revealed significant effects of LPS treatment (*F*_3, 20_ = 1987; *p* < 0.0001) and culture time (*F*_1.756, 35.12_ = 992.4; *p* < 0.0001) with a significant interaction (treatment × time: *F*_9, 60_ = 383.9; *p* < 0.0001). In subsequent Bonferroni post-hoc analyses, the LPS groups with three different doses (10, 100, and 1000 ng/mL) significantly induced higher TNF-α production than the control groups of the three respective periods (6, 12, and 24 h) (Figure 1A). Among the varying doses of LPS, 1000 ng/mL induced microglial TNF-α production over 700 times more than the controls.

Furthermore, we assessed the viability of MG6 cells under LPS stimulation. After 24 h of incubation, the viability of MG6 cells was significantly decreased at 1000 ng/mL (*p* < 0.05), but not at lower doses (10 and 100 ng/mL) (*F*_3, 20_ = 3.744; *p* = 0.0278) (Figure 1B). Since LPS at 100 ng/mL showed a tendency to increase mortality of MG6 (*p* = 0.101), the 10 ng/mL of LPS was used in further experiments.

### 2.2. Effects of NAC on LPS-Induced Cytokine Production in MG6 Cells

To investigate the anti-inflammatory effects of NAC on LPS-induced inflammation, MG6 cells were stimulated with 10 ng/mL LPS alone or in combination with four doses of NAC (5, 10, 20 and 30 mM) for 24 h. One-way ANOVA followed by Bonferroni post-hoc (*F*_4, 25_ = 2.073; *p* < 0.0001) revealed significantly decreased LPS-induced transcript (Bonferroni post-hoc; *p* < 0.05) (*F*_4, 25_ = 10.26; *p* < 0.0001) (Figure 1C), cellular (Bonferroni post-hoc; *p* < 0.05) (*F*_5, 12_ = 17.13; *p* < 0.0001) (Figure 1D), and medium protein (Bonferroni post-hoc; *p* < 0.05) (*F*_4, 25_ = 2.073; *p* < 0.0001) (Figure 1E) levels of TNF-α production in each dose (5, 10, and 20 mM) of NAC compared with LPS exposure alone. The 20 mM NAC inhibited the LPS-induced TNF-α production over 80 times. Although NAC 30 mM significantly reduced the LPS-induced TNF-α protein in the medium (*p* < 0.001; Figure 1E), cellular TNF-α protein was significantly aggregated compared with controls (*p* < 0.05; Figure 1D). Furthermore, real-time PCR and ELISA were used to determine the transcription and protein levels of another major inflammatory cytokine, IL-1β. LPS-induced *Il1b* mRNA (Bonferroni post-hoc; *p* < 0.05) (*F*_4, 25_ = 40.1; *p* < 0.0001) (Figure 1F) and medium protein (Bonferroni post-hoc; *p* < 0.001) (*F*_5, 24_ = 190.2; *p* < 0.0001) (Figure 1G) expression significantly decreased as the NAC dose was increased. Since it is well known that IL-10 inhibits the production of inflammatory cytokines in activated macrophages [46], we determined whether IL-10 is responsible for the antioxidant effects of NAC. We found that only 10 mM NAC significantly increased the transcription of *Il10* (Bonferroni post-hoc; *p* < 0.05) (*F*_4, 25_ = 4.792; *p* = 0.0052) (Figure 1H). The protein levels of IL-10 in the medium under each condition were below the detection limit of the immunosorbent assay (results not shown).

### 2.3. High Concentration of NAC Increased Microglial Mortality without LPS Challenge

**Figure 2:**
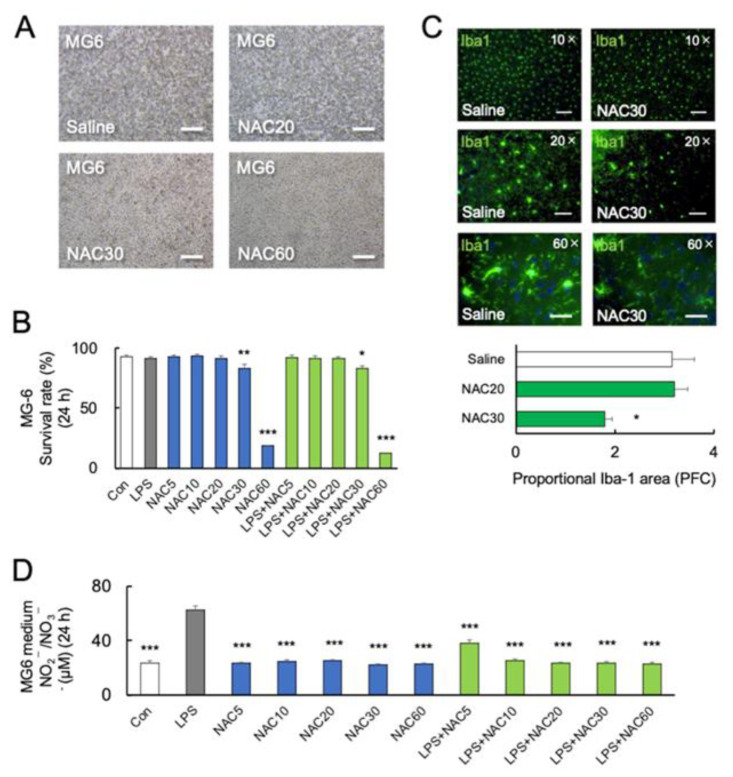
Effects of NAC on LPS-induced MG6 mortality. (**A**,**B**) Representative images and quantitative analyses of MG6 viability at 24 h with different doses of NAC treatments (n = 6). * *p* < 0.05, ** *p* < 0.01, *** *p* < 0.001, vs. control (Con). Scale bars, 250 μm. (**C**) Representative images and quantitative analyses of immunostaining for Iba-1 in the prefrontal cortex of C57BL/6J mice treated with saline or NAC (30 mM) 24 h after the last treatments (n = 4). Iba-1 positive microglia are shown in green. * *p* < 0.05, vs. saline (Con). Scale bars of upper, middle, and lower parts: 100 μm, 50 μm, and 20 μm. (**D**) NO_x_ synthesis after treatment with LPS (0 and 10 ng/mL), and NAC (0, 5, 10, 20, 30, and 60 mM) in MG6 cells after 24 h (n = 6). *** *p* < 0.001, vs. LPS. All data are presented as MEAN ± SEM.

Furthermore, we investigated the effect of NAC and LPS on the viability of microglia. MG6 cells were treated with LPS (10 ng/mL) either alone or in combination with NAC (5, 10, 20, 30, and 60 mM). After 24 h of NAC treatment, a one-way ANOVA followed by Bonferroni post-hoc revealed a significant reduction in viable cells at 30 mM (*p* < 0.05) and 60 mM (*p* < 0.001) NAC (*F*_7, 40_ = 5.277; *p* = 0.01) (Figure 2A,B). Although the levels of LPS-induced inflammatory cytokines were decreased at 30 and 60 mM NAC, a high dose of NAC increased MG6 mortality with LPS (Figure 2A,B). After 24 h coincubation of LPS with NAC at 60 mM, the protein levels of cellular TNF-α were not detected in MG6 by western blotting, according to the highest mortality rate.

Furthermore, C57BL/6J mice were injected intraperitoneally with 20 mM or 30 mM NAC for two days, which is lower than in previous studies [30,47]. On day 3 following treatment with 30 mM NAC, there was a significant decrease in Iba-1 immunoreactivity (*p* < 0.05, Figure 2C) (various magnifying power 10×, 20×, and 60×) in the prefrontal cortex (PFC) compared to the saline group, but not in 20 mM NAC-treated group. Figure 2C shows that following the administration of two doses of 30 mM NAC, the microglial cell bodies in PFC disappeared and their dendritic branches were degraded (60×).

### 2.4. Role of Nitric Oxide in NAC-Induced Microglial Mortality

The inorganic anions nitrite (NO_2_^−^) and nitrate (NO_3_^−^) are end-products of nitric oxide (NO) metabolism [48]. Several studies have demonstrated the potential protective effect of NO [49]. A previous study demonstrated that NAC at a dose of 1000 mg/kg significantly increased the serum NO_2_^−^/NO_3_^−^ in rats [30]. Here, we examined NO synthesis from MG6 cells by measuring NO_x_ (NO_2_^−^/NO_3_^−^). NO_x_ synthesis was significantly increased by LPS (*p* < 0.0001) but not by NAC alone in high concentrations (30 and 60 mM) in comparison to the control (*p* > 0.05) (*F*_11, 36_ = 60.54; *p* < 0.0001) (Figure 2D). Furthermore, NAC (5, 10, 20, 30, and 60 mM) significantly inhibited LPS-induced NO_x_ synthesis (*p* < 0.0001) (Figure 2D), indicating that NO may not be associated with microglial mortality.

### 2.5. Effects of NAC on Acute and Chronic Stress-Induced Behavior

**Figure 3:**
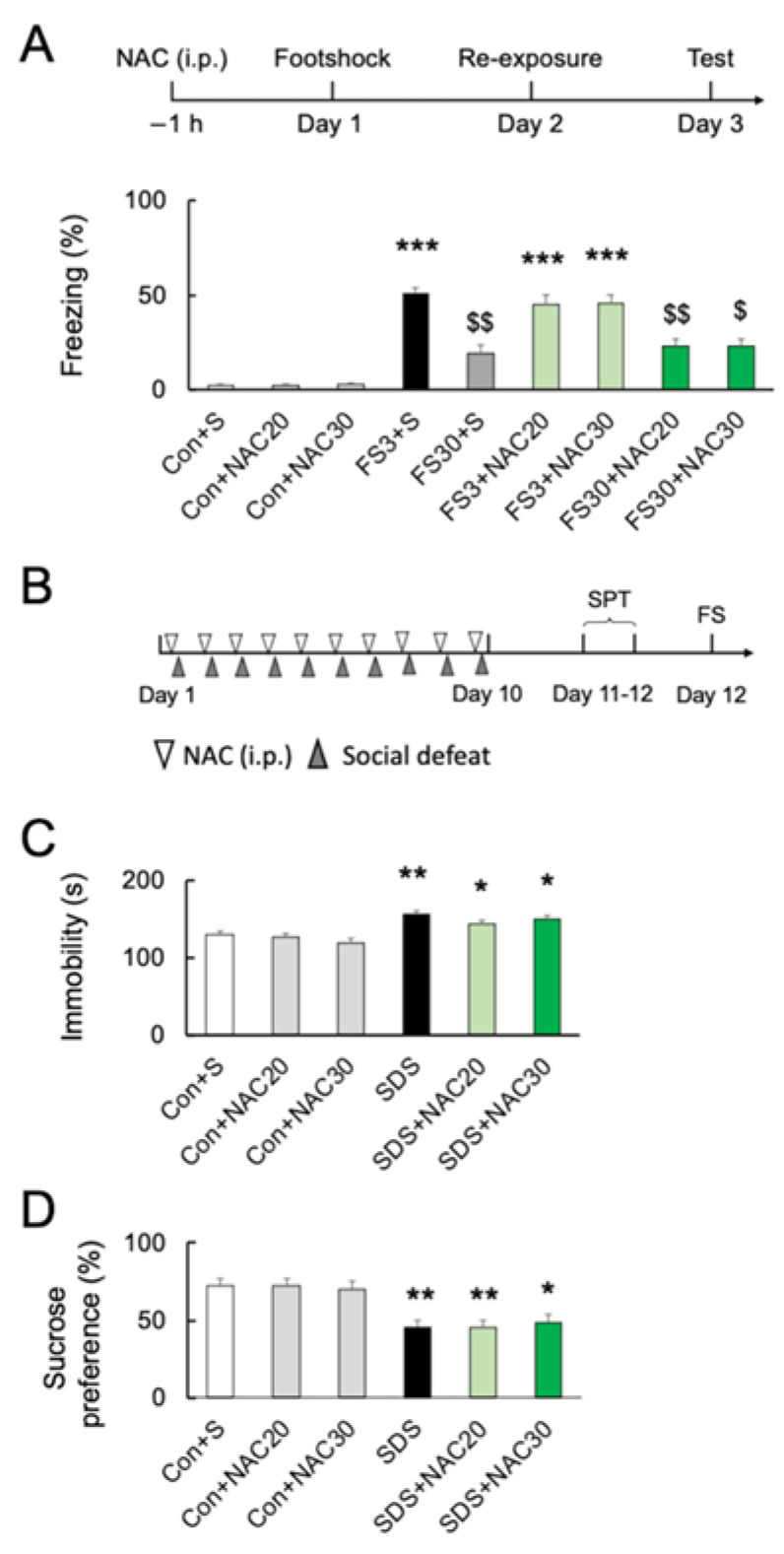
Effects of NAC on acute and chronic stress-induced behaviors. (**A**) Experimental schedule and the effects of NAC (20 and 30 mM) pretreatment on freezing time after acute footshock stress. *** *p* < 0.001, vs. non-stressed control mice. $ *p* < 0.05, $$ *p* < 0.01, vs. FS3 + S. Con, without footshock. FS3, mice after footshock with short-duration re-exposure. S, saline. FS30, mice after footshock with long-duration re-exposure. (**B**) The schedule of the chronic social defeat stress (SDS) experiments. (**C**) The effects of NAC pretreatment (20 and 30 mM) on immobility time in the forced swim test after chronic social defeat stress. * *p* < 0.05, ** *p* < 0.01, vs. non-stressed control mice. (**D**) The effects of NAC pretreatment (20 and 30 mM) on sucrose preference after chronic social defeat stress. * *p* < 0.05, ** *p* < 0.01, vs. non-stressed control mice. All data are presented as MEAN ± SEM.

Although NAC at 30 mM decreased the numbers of microglia in the PFC, pretreatment with NAC at 32.64 mg/kg (20 mM) or 48.96 mg/kg (30 mM) did not affect freezing behavior without footshock (*p* > 0.05; Figure 3A) (*F*_8, 45_ = 34.40; *p* < 0.0001). Acute footshock stress-induced significantly longer freezing times after short-duration re-exposure (FS3) (*p* < 0.001; Figure 3A). In contrast, foot-shocked mice with long-duration re-exposure (FS30) had a significantly longer freezing time than those without footshock (vs. Con; *p* < 0.001) but shorter than re-exposed for a short time after footshock (vs. FS3; *p* < 0.001) (Figure 3A). Pretreatment with 20 mM or 30 mM NAC had no effect on short- or long-duration freezing behaviors (*p* > 0.05) (Figure 3A). Furthermore, we investigated the effect of NAC on chronic social defeat stress (SDS)-induced depressive behaviors in mice (Figure 3B). Following chronic SDS exposure, immobility time in the forced swimming test was significantly longer than in the controls (*p* < 0.01; Figure 3C) (*F*_5, 60_ = 11.63; *p* < 0.0001), and the sucrose preference was decreased as well (*p* < 0.05; Figure 3D) (*F*_5, 54_ = 7.975; *p* < 0.0001). NAC pretreatment did not prevent chronic SDS-induced increased immobility time in forced swimming and decreased sucrose preference at both 20 mM and 30 mM (vs. SDS; *p* > 0.05; Figure 3C,D).

### 2.6. Role of Microglial TNF-α in NAC-Induced Cell Mortality

**Figure 4:**
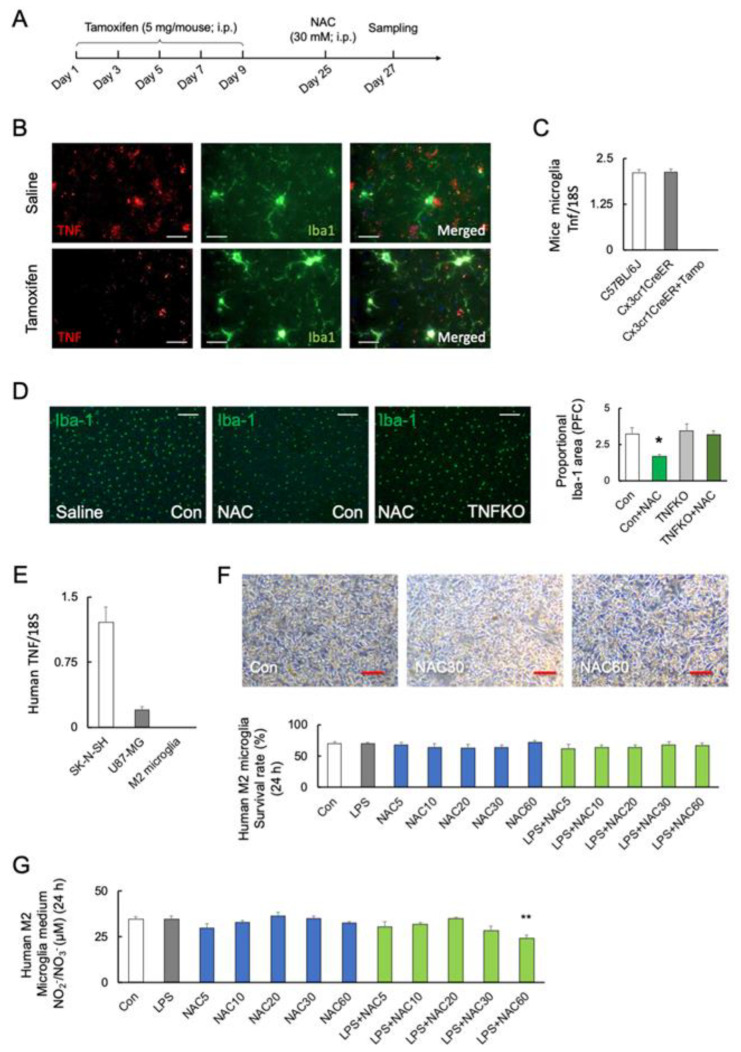
Role of microglial TNF-α in NAC-induced microglia mortality. (**A**) Experimental schedule. (**B**,**C**) Representative images and quantitative analyses of microglial TNF-α in the prefrontal cortex of C57BL/6J and TNF^fl/fl^Cx3cr1-Cre^ER^ mice after saline or tamoxifen treatment. TNF-α and Iba-1 are shown in red and green, respectively. Scale bars, 100 μm. (**C**) The transcript levels of microglial *Tnf* in C57BL/6J mice and TNF^fl/fl^Cx3cr1-Cre^ER^ mice with or without tamoxifen (Tamo) treatment (n = 6). (**D**) Representative images and quantitative analyses of microglia viability after 48 h of NAC (30 mM; i.p.) treatment in C57BL/6J (n = 4) and microglial TNF-α-deficient mice (n = 4) with tamoxifen treatment (TNFKO). * *p* < 0.05, vs. saline (Con; C57BL/6J). Scale bars, 250 μm. (**E**) Transcript levels of *TNF* in the SK-N-SH, U-87 MG, and human primary M2 microglia (n = 4). (**F**) Representative images and quantitative analyses of human M2 primary microglial viability after 24 h of treatment with LPS (0 and 10 ng/mL), and different doses of NAC (0, 5, 10, 20, 30, and 60 mM) (n = 6). Scale bars, 250 μm. (**G**) NO_x_ synthesis after treatment with LPS (0 and 10 ng/mL), and NAC (0, 5, 10, 20, 30, and 60 mM) in human primary M2 microglia after 24 h (n = 6). ** *p* < 0.01, vs. control (Con). All data are presented as MEAN ± SEM.

To investigate the role of microglial TNF-α in microglial mortality, tamoxifen-inducible and microglia-specific TNF-α knockout mice (TNF^fl/fl^Cx3cr1-Cre^ER^) were generated (Figure 4A). The protein levels of TNF-α were decreased after five days of tamoxifen treatment (Student’s *t*-test: *p* < 0.0001; Figure 4B). Figure 4C shows that the Tnf transcripts are undetectable in microglia from tamoxifen-treated TNF^fl/fl^Cx3cr1-Cre^ER^ mice (*F*_2, 15_ = 251.5; *p* < 0.0001). TNF^fl/fl^Cx3cr1-Cre^ER^ mice after tamoxifen treatment (microglial TNF-α KO) significantly prevented the NAC-induced microglia death compared with C57BL/6J (*F*_3, 12_ = 5.235; *p* = 0.015) (Figure 4D).

**Figure 5:**
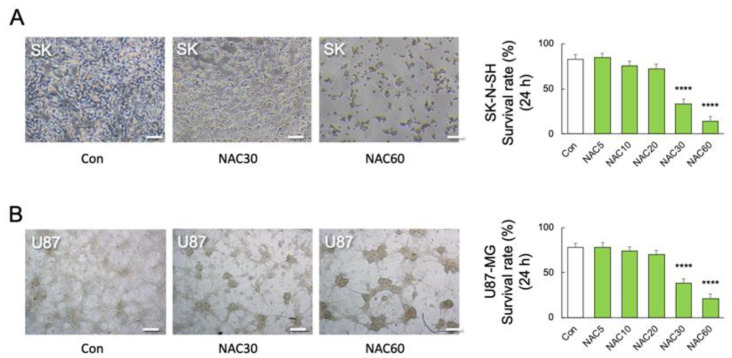
Effects of NAC on LPS-induced mortality in neuronal and astrocytic cells. (**A**) Representative images and quantitative analyses of SK-N-SH (SK) cell viability after 24 h of treatment with different doses of NAC treatments (0, 5, 10, 30, and 60 mM) (n = 6). **** *p* < 0.0001, vs. NAC 0 mM (Con). (**B**) Representative images and quantitative analyses of U-87 MG (U87) cell viability after 24 h of treatment with different doses of NAC (0, 5, 10, 30, and 60 mM) (n = 6). **** *p* < 0.0001, vs. Con. Scale bars, 250 μm. All data are presented as MEAN ± SEM.

We used human primary M2 microglia, SK-N-SH neuroblastoma, and U-87 MG glioblastoma cells to validate the association between TNF-α and NAC-induced cell mortality. Real-time PCR detected TNF transcript levels in SK-N-SH and U-87 MG cells but not in human primary M2 microglia (*F*_2, 18_ = 53.32; *p* < 0.0001) (Figure 4E). The viability of TNF-α-deficient human M2 microglia was unaffected by LPS or NAC alone or in combination (*F*_9, 50_ = 329.3; *p* < 0.0001) (Figure 4F). Furthermore, neither LPS nor NAC induced NO_x_ synthesis in TNF-α-deficient cells but LPS in combination with NAC 60 mM significantly reduced NO_x_ synthesis (*p* < 0.01) (*F*_11, 36_ = 4.101; *p* = 0.0006) (Figure 4G). In contrast, co-stimulation with higher doses of NAC (30 and 60 mM) significantly reduced the viability of human SK-N-SH (*F*_5, 30_ = 39.44; *p* < 0.0001) (Figure 5A) and U-87 MG cells (*F*_5, 30_ = 80.05; *p* < 0.0001) (Figure 5B).

## 3. Discussion

**Figure 6:**
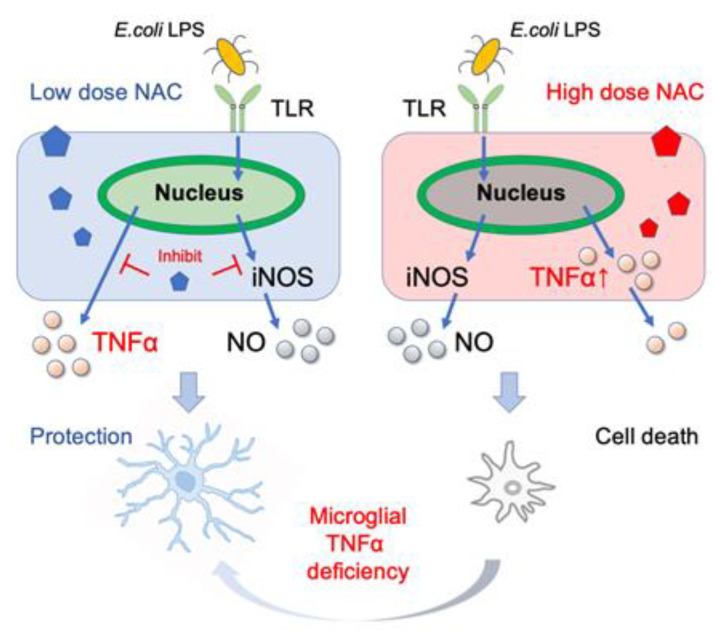
Beneficial and lethal effects of NAC on microglia. The low dose of NAC suppresses the production of *E. coli* LPS-induced TNF-α and NO production (left), whereas high-dose NAC and *E. coli* LPS induce aggregation of cellular TNF-α. Furthermore, microglial TNF-α deficiency alleviates high-dose NAC-induced cell death.

In this study, we tested various doses of NAC for their ability to inhibit LPS-induced synthesis of cytokines and NO in mouse and human microglial cells. LPS significantly increased the transcription of pro-inflammatory cytokines TNF-α and IL-1 but not the anti-inflammatory cytokine IL-10. Moreover, the increased TNF-α and NO levels caused by the low dose of LPS did not affect cellular viability. NAC inhibited LPS-induced TNF-α, IL-1, and NO synthesis in a dose-dependent manner. Low NAC concentrations inhibited LPS-induced TNF-α, IL-1, and NO synthesis in MG6. However, high concentration of NAC (≥30 mM) induced cellular TNF-α aggregation in MG6 and caused cell death in MG6, SK-N-SH, U-87 MG, and PFC microglial cells, but not in human primary M2 microglial and mouse TNF-α-deficient microglial cells (Figure 6).

NAC has been widely used in clinics for over 50 years [50]. NAC is the N-acetyl derivative of the amino acid L-cysteine. It is rapidly absorbed, reaching a peak plasma concentration of 50% after 4 h of oral administration. Due to its short half-life (5.58 h), it is recommended that dosing be divided into two daily doses [51]. L-cysteine rapidly oxidizes to free cystine and regulates glutamate antiporter activity, which is considered the key to therapeutic efficacy in the brain [52]. These effects probably occurred directly in the brain, as NAC crosses the blood–brain barrier and accumulates in the brain [45]. In the majority of previous studies, NAC has been used to inhibit the production of proinflammatory cytokines and reduce cytotoxic levels of NO by inhibiting the synthesis of TNF-α and iNOS. However, the multiple efficacies and toxicological effects of NAC must be highlighted in clinical and animal research. For instance, NAC treatment may induce gastrointestinal and dermatological disorders [41]. NAC treatment can also cause hepatic damage, renal failure, and death in patients with acute carbon tetrachloride poisoning [50]. When compared to controls, NAC treatment (10 mg/kg) for one week after eccentric exercise-induced muscle damage significantly increased tissue damage and oxidative stress [42]. Chronic systemic administration of NAC caused pulmonary arterial hypertension in mice [43]. Continuous infusion of high-dose NAC during a lipopolysaccharide challenge increased mortality in rats [44].

In murine models, recent studies found that pre-exposure to NAC at 2.5 mM for 24 h in N9 microglia cells could entirely prevent the Hg^2+^ -induced transcription of *Tnf*, *Il1b*, and *Nos2* [53]. In vitro, NAC concentrations less than 2.5 mM affected voltage-gated sodium and potassium channels, whereas high doses of NAC inhibited the action potential of rat sciatic nerve fibers [54]. The molecular mechanism by which NAC provides neuroprotection at low doses but causes neurotoxicity at high doses is unknown. According to one theory, a higher dose of NAC could eliminate all the available ROS required for the normal functioning of the cells [54]. However, an in vivo study revealed that pretreatment with 1000 mg/kg NAC elevated serum NO_2_^−^/NO_3_^−^, indicating that NAC has direct scavenging effects on ROS and beneficial effects in sodium taurocholate-induced acute pancreatitis in rats [30]. Our findings revealed that treatment with NAC (≥30 mM) alone had no effect on NO_2_^−^/NO_3_^−^ synthesis but did cause microglia death. Taken together, NO combined with ROS is considered either toxic or protective, depending on the circumstances.

TNF-α is a major proinflammatory cytokine that plays a critical role in both homeostatic and pathophysiological states in epithelial cells, macrophages, microglia, astrocytes, and dendritic cells [55]. Overexpression of TNF-α induces apoptosis and necroptosis in the pathogenesis of inflammatory diseases [56]. Apart from pathogenic effects, TNF has been shown to maintain homeostatic expansion and protect against pathogens. For instance, physiological levels of glial TNF-α are required for synaptic scaling, which adjusts the strength of the synapse [57]. TNF desensitizes macrophages to the deleterious effects of secondary inflammatory challenges through tolerization [58]. In the CNS, TNF-α promotes the proliferation of oligodendrocytes progenitors and neuronal remyelination [59]. TNF-α-deficient displayed improved spatial memory and learning abilities [60,61]. Microglial TNF-α has been implicated in lowering cocaine sensitivity via dopamine receptors [62]. We found that a high dose of NAC (≥30 mM) with LPS reduced medium TNF-α release, whereas increased cellular TNF-α aggregation caused MG6 mortality. Those NAC-induced mortalities were alleviated in TNF-α deficient human and mouse microglia. Here, we present molecular mechanisms underlying the homeostatic functions of microglial TNF-α in NAC-induced cell death that were not associated with NO synthesis (Figure 6). Additionally, NAC (30 mM, i.p.) also caused microglial mortality in the brain without altering acute stress-induced freezing behavior or chronic stress-induced depressive-line behaviors. Although microglia are essential for normal brain development, Cx3cr1-deficient [63] and microglia knockout mice [64] did not exhibit severe abnormal behaviors. Further research should discuss microglia and microglial TNF-α deficiency in regular behavior changes in the future. Taken together, our findings established the effective dose of NAC for protecting inflammation in the CNS and demonstrated that the homeostatic function of TNF-α is associated with NAC-induced microglial mortality.

## 4. Materials and Methods

All experimental procedures were performed in accordance with the Guidelines for the Care of Laboratory Animals of Tohoku University Graduate School of Medicine (Sendai, Japan).

### 4.1. Cell Culture and Cytokine Release

Mice microglia cell line MG6 (RIKEN Cell Bank, Tsukuba, Japan, RRID: CVCL8732) was cultured in Dulbecco’s Modified Eagle Medium (DMEM; GIBCO, Grand Island, NY, USA) supplemented with 10% inactivated fetal bovine serum (FBS; Serana Europe GmbH, Pessin, Germany) at 37 °C in a 5% CO_2_ humidified incubator. Human primary M2 microglia from the cortex were purchased from CELPROGEN Inc. (SKU:37089-01; Torrance, CA, USA). Cells were incubated in a suitable flask at 37 °C in a 5% CO_2_ humidified incubator. Media containing serum of human primary M2 microglia (CELPROGEN, SKU:M37089-01S) was changed every 3 days.

### 4.2. Chemicals and Treatment

To determine the dose–response relationship and time course of *Escherichia coli* (*E. coli*) LPS-induced microglial TNFα production, MG6 cells were treated with LPS from *Escherichia coli* O111:B4 (Sigma-Aldrich, St. Louis, MO, USA, Product Number: L2630) at 0, 10, 100, and 1000 ng/mL to stimulate the release of TNF-α in the cell supernatants were measured by enzyme-linked immunosorbent assay (ELISA) at 0, 6, 12, 24 h, respectively.

Furthermore, MG6, human primary M2 microglia, SK-N-SH, and U87-MG cells were incubated in a medium alone (control) or with 10 ng/mL *E. coli* LPS. The effects of NAC (FUJIFILM Wako, Tokyo, Japan, CAS RN^®^: 616-91-1) (0, 5, 10, 30, and 60 mM) on the viabilities of each cell, transcription, and protein levels of cytokines, and NO productions from MG6 and human primary M2 microglia cells were assessed with coincubation with LPS (10 ng/mL) for 24 h. The non-LPS stimulated group received the same volume of phosphate-buffered saline (GIBCO, Carlsbad, CA, USA). Since NAC is a highly acidic compound (pH 2.2), we adjusted the pH of the NAC solution and culture medium to 8.0.

Tamoxifen (Cayman Ann Arbor, MI, USA) 20 mg were suspended in 100 μL 100% ethanol (FUJIFILM Wako), then dissolved in 900 μL corn oil (FUJIFILM Wako) at a concentration of 20 mg/mL by shaking 95 °C 1 min and 37 °C 1 h [65]. Tamoxifen was injected intraperitoneally (i.p.) for 5 doses of 5 mg with a separation of 48 h between doses. TNF^fl/fl^Cx3cr1-Cre^ER^ mice were i.p. with NAC (20 or 30 mM) for 2 days after 25 days of the last tamoxifen treatment [66].

### 4.3. Animals

All experiments were conducted on male C57BL/6J mice aged 8 to 12 weeks. Mice were purchased from Japan SLC, Inc. (Shizuoka, Japan) and were individually housed and kept on a 12:12 h light/dark cycle with ad libitum access to food and water throughout the experimental period. The animals were acclimated in our animal facility for one week. Microglial TNF-deficient TNF^fl/fl^Cx3cr1-Cre^ER^ mice were used in the current study. Floxed TNFα mice were procured from S. Nedospasov [67] and crossed with Cx3cr1tm2.1(cre/ERT2)Litt/WganJ (RRID:IMSR_JAX:021160)) mouse [68]. TNF^fl/fl^Cx3cr1-Cre^ER^ mice lines were generated at Tohoku University for more than ten generations. After weaning on postnatal days (PNDs) 21–28, all mice were socially housed in same-sexed groups in a temperature-controlled environment with a 12:12 h light/dark cycle (lights on at 09:00 h) with ad libitum access to water and food. Genomic DNA extracted from the tails of mice was used for the standard PCR genotyping.

### 4.4. Acute Stress (Contextual Fear Conditioning Tests)

C57BL/6J mice were given saline or NAC (20 mM or 30 mM, pH 7 in saline; i.p.) 1 h before the first contextual fear conditioning training, at doses similar to those used in vitro experiments (33 or 49 mg/kg), but lower than the previous safety dose (204 mg/kg) [47]. Mice were then placed in the training chamber (17.5 × 17.5 × 15 cm), which had a stainless-steel rod floor that was used to deliver footshocks (Ohara & Co., Ltd., Tokyo, Japan, year 2015). Each mouse was transferred from its home cage to the training chamber (from 10:00 a.m. to 12:00 a.m.) and allowed to explore it for 148 s before receiving a single 2-s footshock (0.4 mA) and consecutive 30-s exposures. Mice were re-exposed to the training chamber without receiving footshocks for 3 min (FS3) or 30 min (FS30) and then transferred to their home cages 24 h after conditioning. FS3 resulted in the retention of fear memory, whereas FS30 facilitated the extinction of fear memory. To validate the effect of NAC on the retention or extinction of fear memory, the percentage of time mice exhibited freezing behavior 24 h after a re-exposure session was measured [21,34]. The percentage of time the mice exhibited freezing behavior during a 5 min exposure (freezing time) was calculated as an indicator of the behavioral outcome of fear memory.

### 4.5. Chronic Social Defeat Stress (SDS)

The chronic SDS procedures were performed as previously described [69]. The clear rectangular cages (26.7 × 48.3 × 15.2 cm) with a clear, perforated plexiglass divider (0.6 × 45.7 × 15.2 cm) (Cat. No. PC10196HT) and paired steel-wire tops (Cat. No. WBL1019 MMB) were purchased from Allentown Inc. (Allentown, NJ, USA). In the chronic SDS session [69], the C57BL/6 mice were treated with saline or NAC (32.64 mg/kg (20 mM) or 48.96 mg/kg (30 mM); pH 7.0; i.p.) per day and were exposed to a different CD1 aggressor mouse for 10 min per day for 10 consecutive days by removing the clear, perforated plexiglass divider. After the last exposure in each session, all C57BL/6 mice were housed individually. The stress-induced behaviors were tested from day 11 to day 12, including the sucrose preference test (days 11–12) and the forced swim test (day 13).

### 4.6. Sucrose Preference Test (SPT)

The SPT was performed as previously described [20]. It employed a two-bottle, free-choice sucrose consumption paradigm. For two days, the mice were habituated to drinking water from two tubes with stoppers fitted with ball-point sippers (Ancare, Bellmore, NY, USA). Following habituation, they were then exposed to 1% sucrose or drinking water for three consecutive days. The weights of the water- or sucrose-containing bottles were measured before and after this period. Sucrose preference was determined using the following equation:

Sucrose preference=(sucrose day 1−sucrose day 2)/((sucrose day 1−sucrose day 2)+(water day 1−water day 2))×100 

### 4.7. Forced Swim Test (FST)

The FST was performed as previously described [70]. Mice were individually placed in an inescapable transparent cylindrical tank filled with water (24 °C) for 6 min. The last 4 min of the test were examined. The behavioral activity was recorded using a video camera. Mobility and immobility times were automatically measured using ANY-Maze video-tracking software Version 6.10 (Stoelting Co., Wood Dale, IL, USA).

### 4.8. Measurement Protein of TNF-α, IL-1, IL-10, Nitric Oxide, and Cell Viability

The microglial culture supernatant stimulated with LPS alone or in combination with NAC was collected, and TNF-α, IL-1β, and IL-10 protein levels were measured using a high-sensitivity enzyme-linked immunosorbent assay kits (kits (BioLegend^®^, San Diego, CA, USA) according to the manufacturer’s instructions, respectively. Nitric oxide levels were measured using a NO_x_ (NO_2_^−^/NO_3_^−^) assay kit (NO_2_/NO_3_ colorimetric assay kit-C II, Dojindo Laboratories, Kumamoto, Japan). TNF-α, IL-1, IL10, and NO levels were measured using a microplate reader SpectraMax^®^ M2e (Molecular Devices, CA, USA) at 450 nm (NO_x_: 540 nm) absorbance. Cell viability was determined using 0.4% trypan blue solution (Sigma-Aldrich, T-8154) and the TC20 Automated Cell Counter (Bio-Rad, Hercules, CA, USA).

### 4.9. Isolation of Microglia

Mice were sacrificed by decapitation, and brains were prepared as a single-cell suspension using a neural tissue dissociation kit (Miltenyi Biotec, Bergisch Gladbach, Germany, 130-093-231) and the gentleMACS™ Dissociator (Miltenyi Biotec, 130-093-235) [21,34]. CD11b-positive microglia were isolated using CD11b-labeled MicroBeads (Miltenyi Biotec, 130-093-634) and the autoMACS^®^ Pro Separator Starter Kit (Miltenyi Biotec, 130-092-545). The purity of CD11b+ cells (>98%) was confirmed using a BD FACSCalibur™ Flow Cytometer (RRID: SCR_000879) (BD Bioscience, Franklin Lakes, NJ, USA) with PE anti-human and anti-mouse CD11b (RRID: AB_2654644) (Miltenyi Biotec, 130–109–363), and stained using rat anti-mouse CD11b FITC-conjugated monoclonal antibodies (130–110–610; Miltenyi Biotec; year 2018) for 5 min at 4 °C. After washing, the cells were subjected to flow cytometry using an ACCURI Flow Cytometer (RRID:SCR_014422) (Accuri Cytometers, Inc., Ann Arbor, MI, USA).

### 4.10. RNA Extraction and Quantitative Real-Time PCR

AllPrep^®^ DNA/RNA/Protein Mini Kit (QIAGEN, Ltd.-UK, Crawley, UK) was used to extract total RNA from MG6, mouse primary microglia, U-87 MG, SK-N-SH, and human primary M2 microglia. SuperScript™ VILO™ cDNA synthesis kit (Invitrogen, Carlsbad, CA, USA) was used to synthesize cDNA. The relative copy number of each transcript in each cDNA sample was determined using specific primers and iQ™ SYBR^®^ Green Supermix (Bio-Rad Inc., Hercules, CA, USA). A standard curve was created for each assay to adjust for differences in the amplification efficiency of the primer sets. 18S rRNA was used as an internal control for normalization. The forward and reverse primers for murine 18S were 5′-GTAACCCGTTGAACCCCATT-3′ and 5′-CCATCCAATCGGTAGTAGCG-3′, respectively. The forward and reverse primers for human 18S were 5′-GAGGATGAGGTGGAACGTGT-3′ and 5′-TCTTCAGTCGCTCCAGGTCT-3′, respectively. The forward and reverse primers for murine Tnf were 5′-AGCCCCCAGTCTGTATCCTT-3′ and 5′-CTCCCTTTGCAGAACTCAGG-3′, respectively. The forward and reverse primers for murine Il1b were 5′-GCCCATCCTCTGTGACTCAT-3′ and 5′-AGGCCACAGGTATTTTGTCG-3′, respectively. The forward and reverse primers for murine IL10 were 5′-CCAAGCCTTATCGGAAATGA-3′ and 5′-TTTTCACAGGGGAGAAATCG-3′, respectively. The forward and reverse primers for human TNF were 5-TCCTTCAGACACCCTCAACC-3 and 5-AGGCCCCAGTTTGAATTCTT-3, respectively.

### 4.11. Cellular Protein Extraction and Western Blotting

AllPrep^®^ DNA/RNA/Protein Mini Kit (QIAGEN) was used to extract total protein from MG6. After measuring protein concentrations by BSA protein Assay Kit (Thermo Fisher Scientific, Cleveland, OH, USA), the same levels of protein from each sample were subjected to sodium dodecyl sulfate polyacrylamide gel electrophoresis (SDS-PAGE; Bio-Rad) and western blotting with the following primary antibodies: polyclonal rabbit anti-TNFα antibody (1:2000; Abcam, ab6671, Cambridge, UK) and monoclonal mouse anti-ACTB antibody (1:1000; Sigma-Aldrich). The secondary antibodies employed were horseradish peroxidase-conjugated anti-rabbit IgG (1:2000; Dako, Glostrup, Denmark) and anti-mouse IgG (1:3000; Jackson ImmunoResearch, West Grove, PA, USA), respectively. Chemiluminescence was detected using an Amersham ECL Plus western blotting detection kit (GE Healthcare, Waukesha, WI, USA) and a ChemiDoc MP Imaging System (Bio-Rad), and the results were quantified using ImageJ 13.0.6 software (http://rsb.info.nih.gov/ij/, accessed on 3 February 2023) [65].

### 4.12. Immunohistochemical Analysis of Mouse Microglia

Immunohistochemistry was performed using a standard protocol [20,21]. In brief, mice were anesthetized with an i.p. injection of Nembutal (pentobarbital sodium, Dainippon Pharmaceutical Co., Ltd., Osaka, Japan) at 0.5 mg/kg and transcardially perfused with phosphate-buffered saline (FUJIFILM Wako), followed by 4% paraformaldehyde phosphate buffer solution (FUJIFILM Wako). The brains were immersed in 4% paraformaldehyde for 24 h before being transferred to a 30% sucrose solution (FUJIFILM Wako) for 24 h. Coronal brain sections of 40 μm thickness were cut using a cryostat (Carl Zeiss MicroImaging GmbH, Jena, Germany) after the brains were rapidly frozen in OCT compound (Sakura Finetek, Torrance, CA, USA). Microglia were detected in the prefrontal cortex (PFC) and hippocampus slices dissected from frozen brains using the rabbit polyclonal anti-mouse ionized calcium-binding adapter molecule 1 (Iba-1) antibody (FUJIFILM Wako, 019-019741). Microglial TNF-α expression was confirmed using the goat polyclonal anti-mouse TNF-α antibody (R&D Systems, Minneapolis, MN, USA. AB-410-NA). The secondary antibodies used were Alexa Fluor™ 488-conjugated anti-rabbit IgG (1:300; Invitrogen, Carlsbad, CA, USA) and Alexa Fluor™ 594 anti-goat IgG (1:300; Invitrogen). The nuclei in the slices were stained with 4′,6-diamidino-2-phenylindole (DAPI; Invitrogen). Cell images were acquired using a fluorescence microscope (Axio Scope.A1; Carl Zeiss, Oberkochen, Germany). The levels of Iba-1 signals were obtained using ImageJ 1.53K software (NIH Image, Bethesda, MD, USA).

### 4.13. Statistical Analysis

All assays were performed on three distinct occasions. Data are expressed as mean ± S.D. For parametric data, all comparisons were made using the Student’s *t* test or one-way ANOVA, followed by a post hoc Turkey’s test. Nonparametric data (ELISA of the primary microglia) were analyzed using the ANOVA test, followed by the Bonferroni test for post hoc analyses. SPSS software version 13.0 (SPSS Inc., Chicago, IL, USA) was used for statistical analyses, and *p* < 0.05 was considered significant.

## Data Availability

The datasets and materials generated and/or analyzed during the current study are available upon reasonable request from the corresponding author.
